# Influence of Mold and Heat Transfer Fluid Materials on the Temperature Distribution of Large Framed Molds in Autoclave Process

**DOI:** 10.3390/ma14154311

**Published:** 2021-08-01

**Authors:** Guowei Zhang, Boming Zhang, Ling Luo, Ting Lin, Xiangchen Xue

**Affiliations:** 1 School of Materials Science and Engineering, Beihang University, Beijing 100191, China; zgwhitbuaa@yeah.net (G.Z.); zbm@buaa.edu.cn (B.Z.); luoling_buaa@yeah.net (L.L.); 2 Design and Development Center, AECC Commercial Aircraft Engine Co., Ltd., Shanghai 201104, China; linting15926487675@163.com; 3 Civil Aviation Division, AVIC COMPOSITE CO., Ltd., Beijing 101300, China

**Keywords:** autoclave process, large framed molds, CFD simulation, temperature performance, process adjustment

## Abstract

Massive composite components manufactured by autoclave curing in large framed molds are extensively used in the aerospace industry. The high temperature performance of the large framed mold is the key to achieving the desired composite part quality. This paper explores and summarizes the important thermal properties of metal and heat transfer fluid materials influencing the heating performance of large framed molds, with the aim of improving the mold temperature distribution. Considering the fluid–thermal–solid interaction inside the autoclave, a reliable computational fluid dynamics (CFD) simulation model was developed and verified by a temperature monitoring experiment to achieve the prediction of the temperature distribution of the large framed mold. Then, numerical simulations were designed on the basis of the CFD model, and the single-variable method was used to study the effects of the material thermal properties on the temperature performance of large framed molds. Our simulation predicts that when copper is used as the mold material, the temperature difference decreases by 30.63% relative to that for steel, and the heating rate increases by 3.45%. Further, when helium is used as the heat transfer medium, the temperature difference decreases by 68.27% relative to that for air, and the heating rate increases by 32.76%. This paper provides a reference for improvement of large framed mold manufacturing and autoclave process in terms of heating rate and temperature uniformity.

## 1. Introduction

Fiber-reinforced polymer composites with excellent mechanical properties, low density, and designability are widely used in the aviation, aerospace, and transportation fields. The autoclave curing process is an indispensable manufacturing method for producing large and high-performance composite components, such as wings and fuselages [1,2]. In the autoclave process, temperature and pressure are two vital factors that have a significant influence on the mechanical properties of the composites [3,4,5]. As the curing temperature increases, thermoset composites typically go through three stages: liquid, rubber, and solid [6]. Generally, void formation and uneven resin flow will appear in the liquid and rubber stages owing to the non-uniform temperature field distribution of the composites in the autoclave process [7,8]. Therefore, it is of significant importance to promote the temperature field distribution of the composite parts to ensure product quality. Convective heat transfer and thermal conduction are two important heat transfer modes in the autoclave process [9,10,11]. As the composite component size increases, the mold has a significant influence on the manufacturing of the component owing to the large thermal mass compared with that of the composite part [12,13,14,15]. Therefore, in the autoclave process, the temperature field distribution of the component and the mold are closely related. Mold temperature uniformity determines the achievement of the final composite component quality. However, a large framed mold, which has the characteristics of a huge mass and structural complexity, results in a low heating rate. Additionally, the nonuniform temperature field is determined by the variation in the fluid velocity in the autoclave process. An excessive temperature is an adverse factor to affect the curing of the composite component, forming residual stresses and strains, which weakens the final component quality [16,17]. Therefore, improving the heating rate and temperature uniformity of the large framed mold plays a crucial role in ensuring the composite part quality.

It is difficult and time-consuming to improve the temperature uniformity of a large composite part and mold by adopting a trial-and-error method. With the development of computers, process modeling simulation has come to be regarded as an efficient and low-cost tool, which can be combined with experiments to establish a reliable model. Numerical simulations have been extensively used to improve the temperature field of composite parts in the autoclave process. Kim and Lee [18] simulated the temperature field distribution of thick laminates and proposed an optimized temperature cycle for the laminates to reduce overheating during curing. Park et al. [19] proposed a finite element model for the prediction of the temperature at any point within a composite structure. Gopal et al. [20] reported a numerical simulation method that identifies the gradients resulting from applied temperatures at different dwell times as process parameters. They also proposed an optimal cycle that reduces the residual stresses and curing time. Guo et al. [21] implemented nonlinear transient heat transfer finite element codes for simulating the curing processes of thick composite laminates to reduce the out-of-plane temperature gradient. Aleksendrić et al. [22] developed an optimization model using genetic algorithms to improve the degree of curing and temperature distribution of composites. Struzziero et al. [23] established an optimization model that combined the finite method and genetic algorithm for thick composite components to lessen the temperature overgrow and curing time. In these studies, the curing cycle temperature was applied evenly to the surface of the composite component as a uniform boundary condition, while the influence of the mold and surrounding fluid on the temperature was not considered. These simulation methods are more appropriate for small-scale and uncomplicated composite components. However, in the autoclave process, the effect of the mold and fluid on the heat distribution of the composite components is nonnegligible.

Compared with the extensive research on the temperature field distribution of composite components, reports on investigations of the thermal performance of molds are rare. As computer technology develops, computational fluid dynamics (CFD) has been applied to simulate the autoclave process. Weber et al. [24] simulated the heat transfer phenomenon in the autoclave from the thermal point of view and pointed out that the displacement factor determined the heat transfer coefficients of different regions. Their research proposed a semi-empirical method for the estimation of thermal boundary conditions. Slesinger et al. [25] simulated and reported that the heat transfer coefficients of different positions around the mold are different, and the heat transfer coefficients differ by as much as 2–3 times. Hudek et al. [26] simulated the heat transfer in the autoclave and proved that the factors affecting the heat transfer in the autoclave included the vacuum bag, the position of the mold, and the direction of the heat flow. Duval et al. [27] simulated the transient thermal distribution of the mold and verified the reliability of the simulation data by comparing the results of the experiment and simulation. Vafayan et al. [11] used Abaqus and MATLAB software to simulate the heat distribution of complex composite components, studied the relationship between curing temperature and time, and found the best curing cycle for composite part curing. Gao et al. [28] simulated the heating process in the autoclave with numerical calculation methods. In their research, the flow field and temperature field distribution in the autoclave were obtained, and the accuracy of the simulation results was verified by comparison with experimental data. Yue et al. [29] simulated the influence of mold heat distribution on the deformation of composite components and pointed out the significance of studying mold temperature field distribution. Zhang et al. [30] studied the influence of various process conditions and environmental parameters on the heat distribution in the autoclave and proposed a simplified model for the heat transfer analysis in the autoclave. Kluge et al. [31,32] conducted an experiment and a CFD simulation to propose that proper inlet boundary conditions were important for mold temperature prediction. Chen et al. [33,34] conducted a CFD simulation that was in good agreement with experimental observations. They also proposed that the K-epsilon turbulence model and unstructured mesh were satisfactory for CFD simulation. Xie et al. [15] developed a CFD simulation method to predict the thermal distribution of a framed mold and demonstrated that in contrast with the external temperature field, the curing exothermic reaction did not have a significant influence on the temperature distribution. Dolkun et al. [16] combined CFD simulation with a response surface methodology to discuss the influence of large framed mold placement on temperature uniformity. Wang et al. [35,36] optimized substructures of a mold using a method that combined numerical simulation with a genetic algorithm for improving temperature uniformity in the curing process. Han et al. [37] reported that changing the framed mold placement angle in the autoclave led to a significant improvement in the temperature uniformity of the mold. However, research on the influence of mold and heat transfer fluid materials on the heating performance of large framed molds has rarely been reported in the open literature. The thermal properties of the mold and heat transfer fluid materials have a significant influence on the temperature performance of the large framed mold. Exploring the effect of different combinations of mold materials (such as steel, copper, and aluminum) and fluid materials (such as air, helium, and argon) on the heating performance of large framed molds is of significant importance for mold manufacturing and process adjustment.

This study aimed to improve the heating performance, including both the heating rate as well as temperature uniformity, of large framed molds in terms of the interaction between the mold and fluid materials. To this end, a CFD simulation model based on the finite volume method was developed for large framed molds in an autoclave. An experiment was carried out for monitoring the temperature distribution of the large framed mold panel to validate the CFD simulation model. To discuss the influence of the thermal properties of the materials on the heating performance of large framed molds, numerical simulations were designed based on the validated CFD model using the single-variable method. Finally, the effects of different combinations of commonly used mold and fluid materials on heating performance were also estimated. This paper provides suggestions and references for manufacturing large framed molds and for optimizing the autoclave process in terms of the heating rate and temperature uniformity.

## 2. Experiment

### 2.1. Temperature Monitoring Experiment

**Figure 1:**
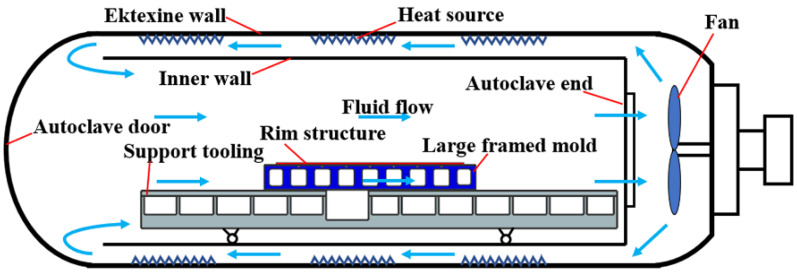
Schematic diagram of the autoclave.

A full-size industrial autoclave (produced in Zhucheng Luguantong Machinery Technology CO., Ltd., Weifang, China) with a cylindrical inner cavity, 3.7 m in diameter and 9.6 m in length, was used for the temperature monitoring experiment. The relative positions of the structures in the autoclave are shown in Figure 1, including bottom support tooling, large framed mold, and rim structure that is used to limit the shape of the curing composite part. Air and nitrogen are commonly used heat transfer fluids in autoclaves. The heated fluid was circulated by a fan, which ensured forced convective heat transfer when the fluid passed through the mold. The length, width, and height of the large framed mold were 4000 mm, 3200 mm, and 480 mm, respectively, and the length between the mold and the autoclave end was 3015 mm. The large framed mold was made of Q345 steel, and air was used as the heat transfer fluid.

**Figure 2:**
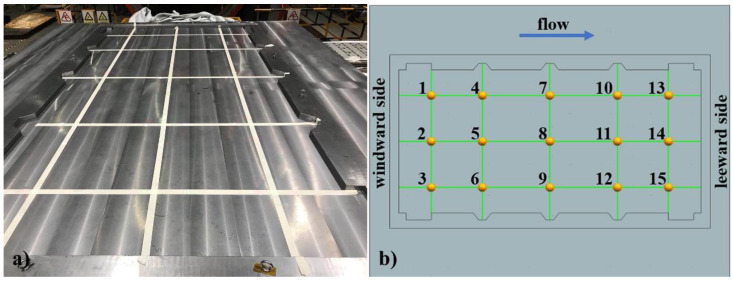
(**a**) Large framed mold; (**b**) Distribution of thermocouples.

The distribution of thermocouples is illustrated in Figure 2. Fifteen thermocouples (produced in Shanghai E-B Automation Instrument CO., Ltd., Shanghai, China) (type k, accuracy ±0.1 °C) were fixed within the rim on the large framed mold plate to obtain the temperature distribution data. Temperatures were recorded at intervals of 1 min. Points 1, 2, and 3 were arranged on the windward side near the autoclave door. Points 13, 14, and 15 were arranged on the leeward side, close to the autoclave end.

**Figure 3:**
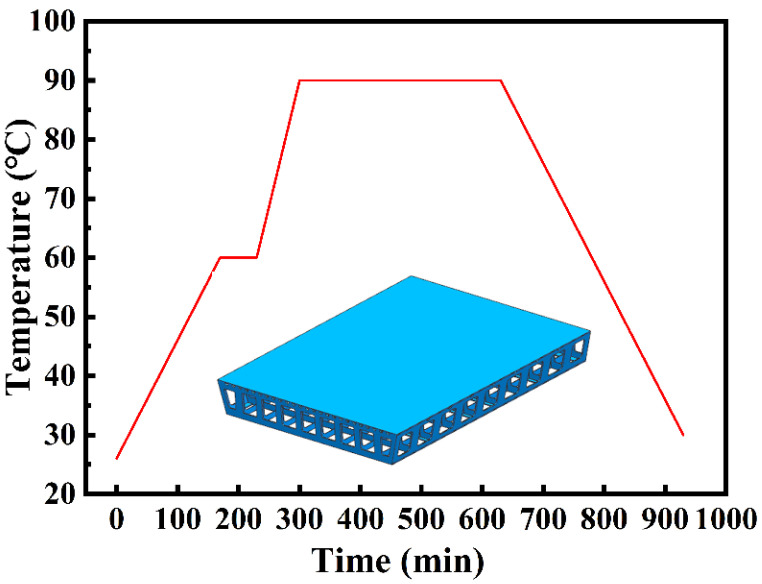
The curing cycle curve.

Figure 3 shows the curing cycle curve. It includes two heating stages, two holding stages, and a cooling stage. In the first stage, it took 170 min for the temperature to rise from 26 to 60 °C, following which the temperature was maintained at 60 °C for 60 min. In the second stage, the temperature raised from 60 to 90 °C in 70 min, after which the temperature was maintained at 90 °C for 5.5 h. The duration of the cooling stage was 5 h, and the cooling rate was 0.2 °C/min. In the experiment, the pressure was set to 0.2 MPa at a starting temperature of 45 °C. The pressure was released after the cooling stage. To reduce the cost of the experiment, temperature and pressure history data were recorded only during the heating and holding stages, as the data for these stages were sufficient to determine the CFD simulation model.

### 2.2. Experimental Results

**Figure 4:**
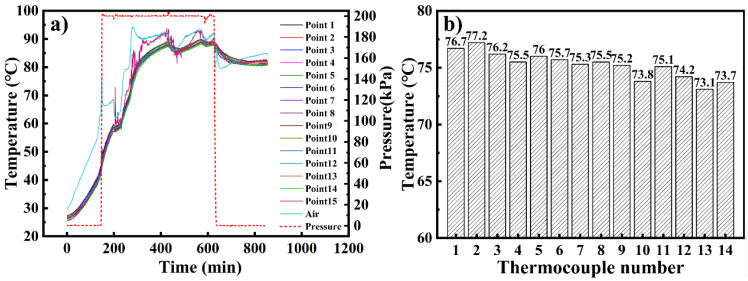
Temperature history (**a**) for the entire process and (**b**) at 300 min.

Figure 4a shows the temperature at the fifteen monitoring points and the air during the curing process. In the heating stage, the air temperature was always higher than that of the mold panel. The different temperatures and heating rates were determined by the variation in flow velocity of the air through the mold. Figure 4a also shows that when the autoclave was pressurized at t = 150 min, the heating rate at each point increased, reflecting the influence of the operating pressure on the heating rate [38]. The abnormal fluctuation observed at point 15 was due to the failure of the thermocouple; owing to this, point 15 was excluded from the subsequent discussion. Figure 4b shows the temperature data at the end of the second heating stage (t = 300 min), when the maximum temperature difference was observed. After 300 min, the temperature uniformity of the mold was improved by the holding stage. The highest temperature was recorded at point 2, close to the windward side, while the lowest temperature was recorded at point 13 on the leeward side.

**Figure 5:**
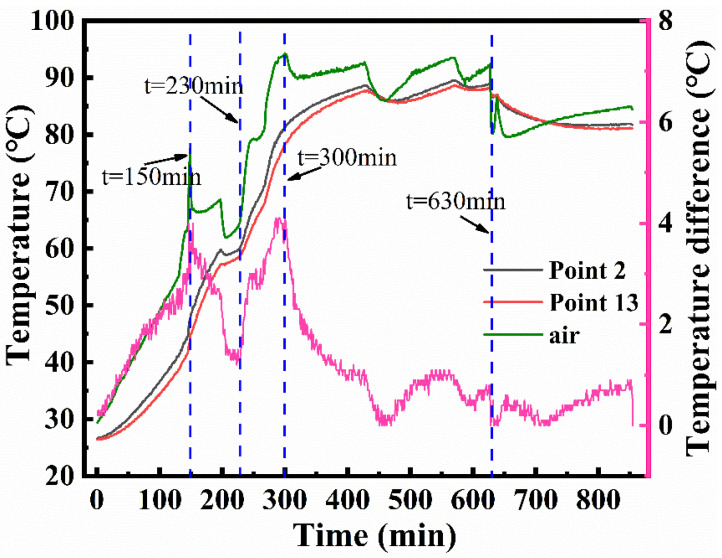
The maximum temperature difference.

The temperature difference between point 2 and point 13 is shown in Figure 5. The temperature difference experienced several large fluctuations. The first peak on the curve was caused by initial pressure applied at t = 150 min, implying that the pressure affected not only the heating rate but also the temperature uniformity of the large framed mold. The maximum temperature difference (4.1 °C) was observed at the time t = 300 min, whereas points 2 and 13 did not reach the maximum temperature. In the two holding stages, the temperature difference gradually decreased. The trough that appears during the holding stage was caused by the temperature drop of the autoclave control system, which was allowed within a certain range.

## 3. Simulation and Validation

### 3.1. Governing Equations

Fluid flow and heat transfer phenomena occur during the autoclave curing process [15], and they are all governed by three basic physical laws—namely, conservation of mass, momentum, and energy [26,34].

Mass conservation equation:

(1) $$\frac{\partial \rho }{\partial t}+div\left(\rho U\right)=0$$

Momentum conservation equation:

(2) $$\frac{\partial \left(\rho U\right)}{\partial t}+div\left(\rho U⊗U\right)=div\left(\mu \;grad\;U\right)-grad\;p+S$$

where *ρ*, *µ*, and *p* are the density, dynamic viscosity, and pressure of the fluid, respectively; *S* is the generalized source term of the momentum equation, and **U** is the velocity component in the x, y, and z directions.

Energy conservation equation:

(3) $$\frac{\partial \left(\rho h\right)}{\partial t}+div\left(\rho hU\right)=div\left(\lambda \;grad\;T\right)-pdiv\left(U\right)+S_{h}+\varphi$$

where *h*, *λ*, *T*, and *S_h_* are the fluid enthalpy, thermal conductivity, temperature, and internal heat source of the fluid, respectively, and *φ* is the dissipation function.

The three basic equations cannot be sufficient; therefore, it is necessary to supplement other state equations. The equation of a gas state can be represented as:

(4) ρ=f(p,T)

There is only heat conduction in the solid region. The heat conduction equation is expressed as follows:

(5) $$\rho_{s}c_{s}\frac{\partial T}{\partial t}=div\left(\lambda_{s}div\left(T\right)\right)+S_{T}$$

where *ρ_s_*, *c_s_*, *λ_s_*, and *T* are the density, specific heat, thermal conductivity, and temperature of the solid, respectively, and *S_T_* represents the internal heat.

The autoclave process involves forced convection heat transfer; therefore, it is crucial to determine the fluid type. Generally, the flow type can be either laminar or turbulent, and flow is determined by the Reynolds number, *R*_e_, which is defined as follows:

(6) $$R_{e}=\frac{\rho v_{m}D}{\mu }$$

where *ρ*, *ν_m_*, *D*, and *µ* are the density, velocity, region diameter, and dynamic viscosity of the fluid, respectively. The value *R*_e_ of turbulent flow is larger than 12,000 [34]. The values of these properties for air, used as the heating fluid in the experiment, are as follows: *ρ* = 1.204 kg/m^3^, *ν_m_* = 2.5 m/s, *D* = 3.7 m, and *µ* = 18.5 × 10^−^^6^ kg/(m·s). *R*_e_ was calculated to be 602,000. Therefore, the flow type in this experiment was turbulent flow.

### 3.2. CFD Model Establishment

**Figure 6:**
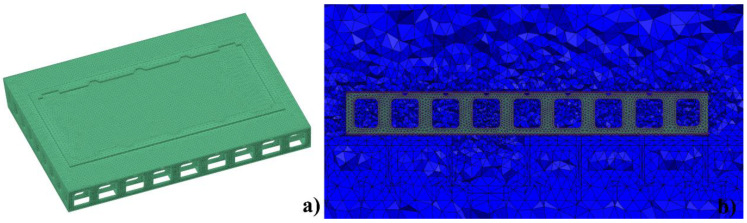
(**a**) Mesh of the mold; (**b**) Fluid–solid coupled mesh.

A CFD simulation model based on the previous convective heat transfer model was developed for the large framed mold in the autoclave. The finite element software Simcenter (version 12.0), developed by Siemens, was used for the numerical simulation. A K-epsilon turbulence model was selected for the simulation in view of its accuracy and computational cost. Owing to the complex structure of the large framed mold, an unstructured mesh was applied in the simulation. There were 4,127,894 tetrahedral elements and 724,877 nodes in the CFD model. The calculation time for one case was 28 h. Figure 6a shows the mesh of the large framed mold. As shown in Figure 6b, the grid size gradually increased from the solid region (green) to the fluid region (blue). The mold material was Q345 steel. The material properties were as follows: density, 7829 kg/m^3^, specific heat, 434 J/(kg∙°C), and thermal conductivity, 54.6 W/(m∙K).

**Figure 7:**
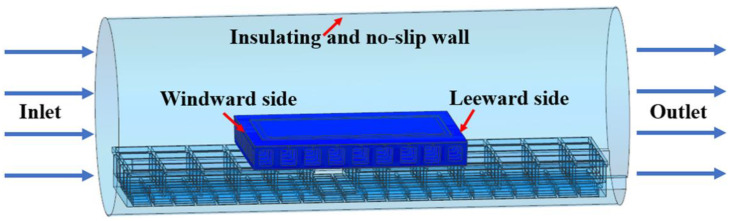
Boundary conditions.

The simplified boundary conditions are shown in Figure 7. The effective calculation region in the autoclave was a cylindrical cavity with an insulating and no-slip wall [39]. The temperature boundary condition was set at the inlet, which was the curing temperature curve. The convergence criterion of the residual value was set to less than 10^−5^ [40]. The simulation time step was 5 min.

### 3.3. Model Validation

**Figure 8:**
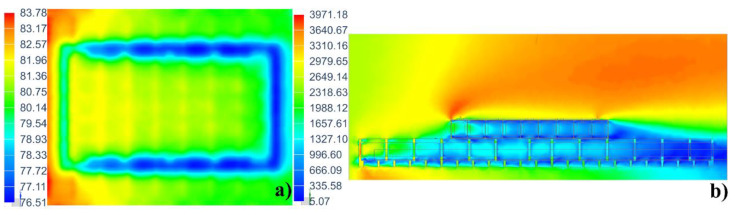
Contour plot of (**a**) temperature field distribution of the mold (unit: °C) and (**b**) velocity field distribution of the air (unit: mm/s).

The temperature field distribution of the mold plate and the velocity field distribution of the air at t = 300 min are shown in Figure 8. The temperature close to the autoclave door is significantly higher than that close to the autoclave end, which is consistent with the experimental results (Figure 4). This is because the frame structure of the mold hindered the circulation of hot air and affected the efficiency of convective heat transfer. Concurrently, the influence of the rim structure on the temperature field is also obvious [16].

**Figure 9:**
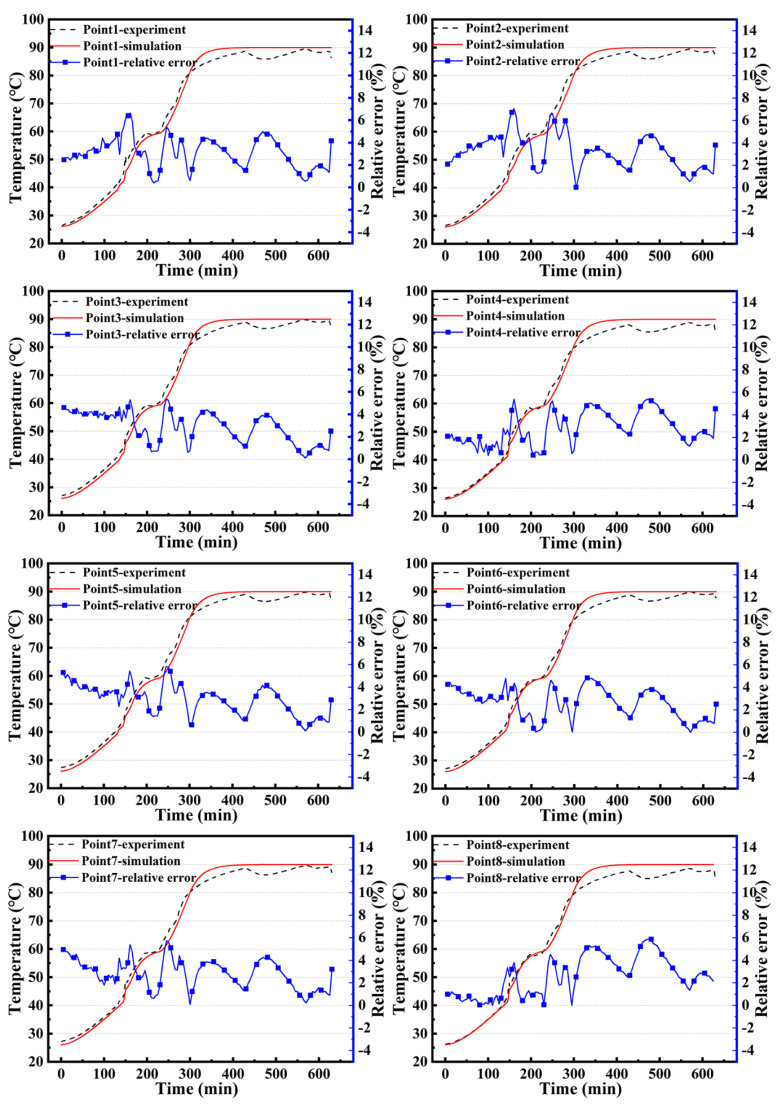

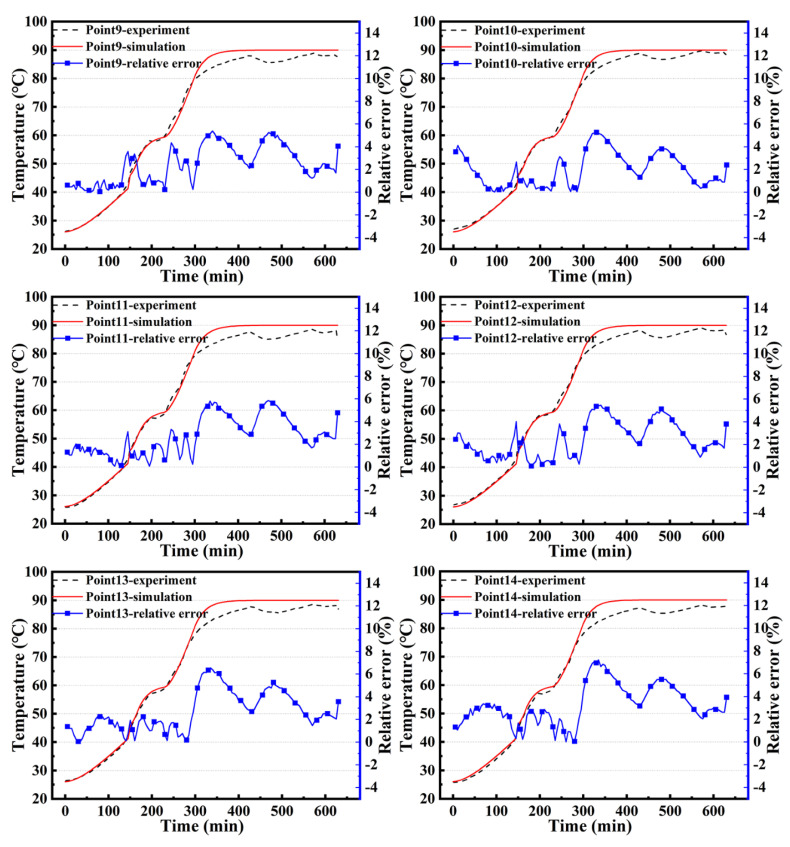
Comparison and relative errors between simulation results and measured temperatures at the fourteen points.

Figure 9 shows a comparison and relative errors between the simulation results and measured temperatures at the fourteen points. It is clear that the temperature evolution trends for the fourteen points are similar.

**Figure 10:**
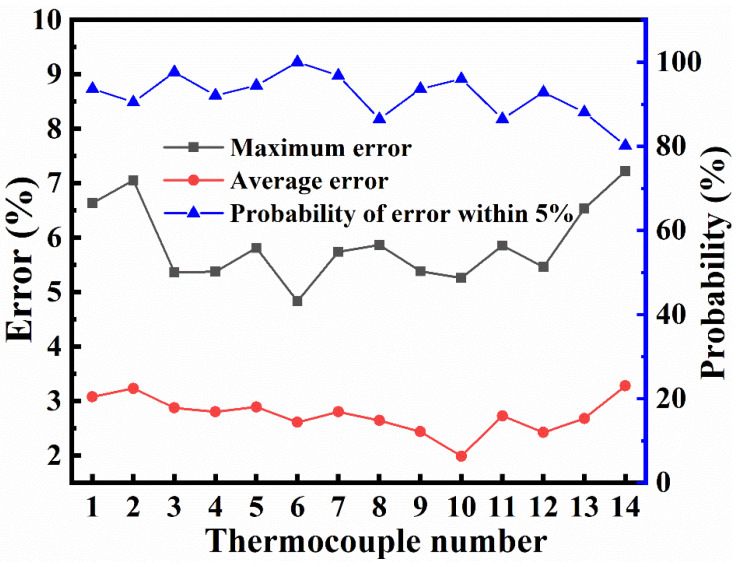
Simulation error distribution.

To compare the simulation and experimental data, simulation errors were counted. The total time of the heating and holding stages was 630 min, and the simulation time step was 5 min; therefore, there were 126 time points for comparison with the experiment in the simulation. Comparing the simulation with the experiment, the maximum and average errors both appear at point 14, which are 7.22% and 3.28%, respectively. The probability of the time points at which the error of each thermocouple point was within 5% is shown in Figure 10. The lowest probability appears at point 14, and more than 80% of the time points are within a margin of 5%. The highest probability occurs at point 6, and every time point is within a 5% margin; therefore, the CFD model determined by the experiment is considered to be effective.

## 4. Design of Simulations

After establishing the effectiveness of the CFD model, numerical simulations were designed for a framed mold to elucidate the effect of metal and fluid materials on the heating performance and to identify preferable materials to ensure temperature uniformity and heating rate. To investigate the effects of different material variables on the temperature behavior of the large mold, we adopted the single-variable method for performing the numerical simulations. The temperature distribution of the large framed mold was controlled by four thermal variables, i.e., the specific heat capacities and thermal conductivities of the mold and fluid. It is crucial to evaluate the mold temperature performance. The additional structures, such as rim structures, were not considered in the simulations. The temperature uniformity of the mold was characterized by the maximum temperature difference (TD_max_) [37], which is defined as follows:

(7) $${\mathrm{TD}}_{\max}=\left|T_{\max}-T_{\min}\right|$$

where T_max_ and T_min_ are the maximum and minimum temperatures of the large framed mold plate, respectively. It is impossible to eliminate temperature differences (TD) in a large framed mold owing to its structural complexity. Therefore, minimization of the TD is important to promote the temperature conformity of the mold.

The objective of this study was to explore the influence of different combinations of mold and fluid materials on the heating performance of large framed molds. Common metals that are used to manufacture molds and their thermal properties are listed in Table 1. Common non-toxic, harmless, and low-cost inert heat transfer fluids and their thermal properties are listed in Table 2. The material property data were obtained from the Siemens General Materials Database.

As shown in Table 1, the thermal conductivities of the metals are in the range of 14–387 W/(m∙K), and the specific heat capacities are in the range of 385–963 J/(kg∙°C). Table 2 shows that the thermal conductivities and specific heat capacities of the fluid materials are in the range of 0.0154–0.152 W/(m∙K) and 900–5193 J/(kg∙°C), respectively. Herein, to explore the influence of different thermal parameters of materials on the temperature performance of large framed molds, the single-variable method was used for simulations. On the basis of Q345 steel and air, the single-thermal parameter was changed to exclude the influence of other thermal properties. The selection range of the simulation parameters included the range of thermal parameters for each material. Five values were selected for each material thermal parameter range in Table 3.

After studying the effect of material thermal properties on the temperature performance of the framed mold, the effect of different combinations of mold and fluid materials was investigated. The different material combinations are listed in Table 4.

## 5. Results and Discussion

**Figure 11:**
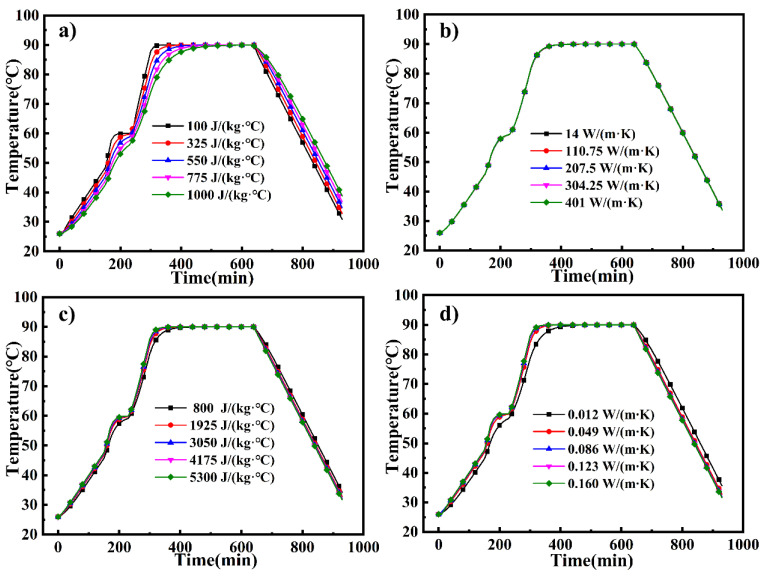
Average temperature of the mold plate as a function of (**a**) specific heat of the mold, (**b**) thermal conductivity of the mold, (**c**) specific heat of the fluid, and (**d**) thermal conductivity of the fluid.

The average temperatures (T_avg_) of the large framed mold plate for different thermal parameters are shown in Figure 11. Here, the average pertains to 10,313 nodes on the mold panel. All the temperature curves exhibit a similar trend, which is consistent with the curing curve. In the two heating stages (t = 0–170 min and t = 230–300 min), the temperature of the mold increases gradually, but a sudden increase in the heating rate occurs at t = 150 min, owing to the application of pressure, which was corroborated by the experiment. In the two holding stages (t = 170–230 min and t = 300–630 min), the rate of temperature increase decreases gradually with time [33]. Finally, in the cooling phase (t = 630–930 min), the temperature decreases gradually. As the two heating stages are governed by the same laws, in the subsequent discussion, we will focus on the temperature and heating rate of the second heating stage. The ending time (t = 300 min) of the second heating phase is a crucial observation and will be discussed later.

**Figure 12:**
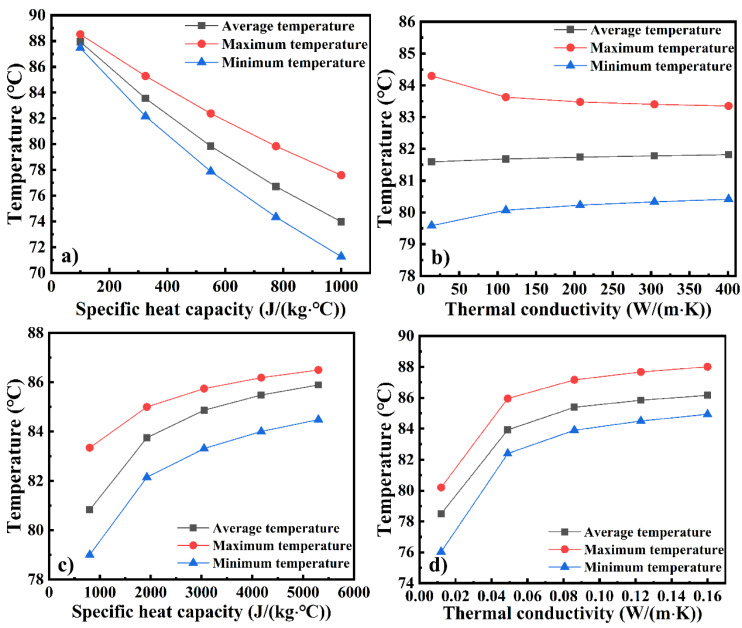
The average, maximum, and minimum temperature of the mold plate as functions of (**a**) specific heat of the mold, (**b**) thermal conductivity of the mold, (**c**) specific heat of the fluid, and (**d**) thermal conductivity of the fluid.

Figure 11a shows that the T_avg_ of the mold plate decreases with an increase in the specific heat of the mold material. The average heating rate of the mold decreases from 0.458 °C/min to 0.275 °C/min. This is because a higher specific heat capacity leads to a slower rise in the mold temperature. As shown in Figure 11b, the T_avg_ of the mold increases with the thermal conductivity of the mold, where there is a slight difference between the temperature values, as shown in Figure 12b, and the average heating rate of the mold raises from 0.347 °C/min to 0.350 °C/min. Figure 11c displays that the T_avg_ of the mold in the heating stage increases with the specific heat capacity of the fluid, and the average heating rate of the mold increases from 0.339 °C/min to 0.398 °C/min. The T_avg_ of the mold increases with the thermal conductivity of the fluid, and the average heating rate of the mold raises from 0.314 °C/min to 0.402 °C/min, as is shown in Figure 11d.

In order to understand the temperature evolution in depth, the T_avg_, T_max_, and T_min_ of the large framed mold plate at t = 300 min were studied as functions of different thermal parameters, and the results are shown in Figure 12. Figure 12a shows that with an increase in the specific heat capacity of the mold, the T_avg_, T_max_, and T_min_ of the mold decrease, and the TD of the mold increases. The T_avg_ decreases from 87.94 °C to 73.96 °C (reduced by 15.89%). Figure 12b shows that with an increase in the thermal conductivity of the mold, the T_avg_ and T_min_ of the mold surface increase, but the T_max_ and TD decrease, which indicates that the heat uniformity of the mold improved. This is also consistent with the slight change in the T_avg_ and heating rate of the mold panel, as shown in Figure 11b. Therefore, although the thermal conductivity of the mold had only a minor effect on the temperature and heating rate, it had a significant effect on temperature distribution; the T_avg_ increases from 80.59 °C to 81.82 °C (an increase of 0.27%) as the thermal conductivity of the mold increases over the range of values studied. Figure 12c shows that with an increase in the specific heat capacity of the fluid, the T_avg_, T_max_, and T_min_ of the large framed mold increase, while the TD of the mold decreases and the T_avg_ increases from 80.83 °C to 85.89 °C (an increase of 6.27%). Figure 12d shows that with an increase in the thermal conductivity of the fluid, the T_avg_, T_max_, and T_min_ of the mold panel increase, while the TD of the mold decreases. The T_avg_ increases from 78.49 °C to 86.15 °C (an increase of 9.76%).

**Figure 13:**
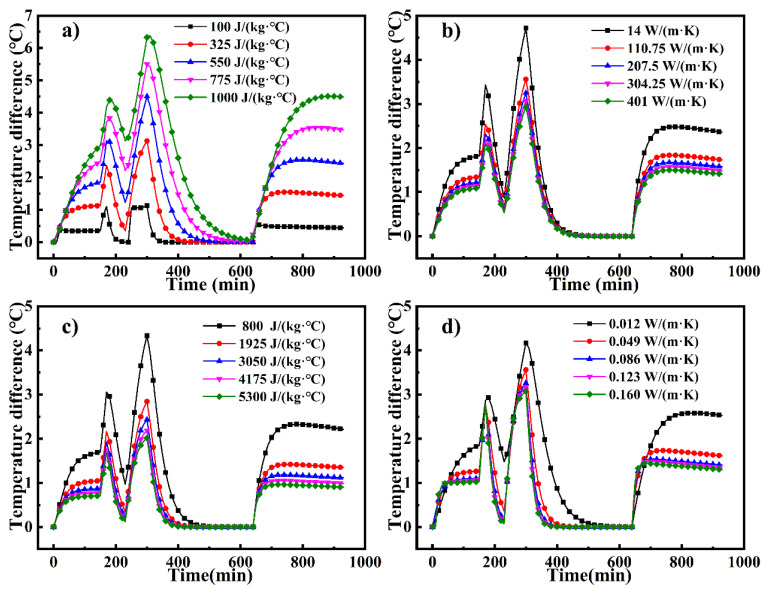
Maximum temperature difference on the mold plate as a function of (**a**) specific heat of the mold, (**b**) thermal conductivity of the mold, (**c**) specific heat of the fluid, and (**d**) thermal conductivity of the fluid.

The TD_max_ on the large framed mold panel as a function of different thermal parameters is shown in Figure 13. The TD_max_ increases in the heating stage and decreases in the holding stage. At the ending time of the second heating phase (t = 300 min), the TD_max_ reaches its maximum value [33]. Figure 13a shows that as the specific heat capacity of the mold increases in the range of values studied, the maximum TD_max_ of the large framed mold panel gradually increases from 1.06 °C to 6.33 °C. Figure 13b shows that as the thermal conductivity of the mold increases in the range studied, the maximum TD_max_ of the mold gradually decreases from 4.71 °C to 2.93 °C. Figure 13c shows that as the specific heat capacity of the fluid increases over the range of values studied, the maximum TD_max_ of the mold decreases from 4.34 °C to 2.02 °C. Figure 13d shows that as the thermal conductivity of the fluid increases over the range studied, the maximum TD_max_ of the mold decreases from 4.17 °C to 3.07 °C. As the TD_max_ decreases, the temperature of the mold plate becomes more uniform.

**Figure 14:**
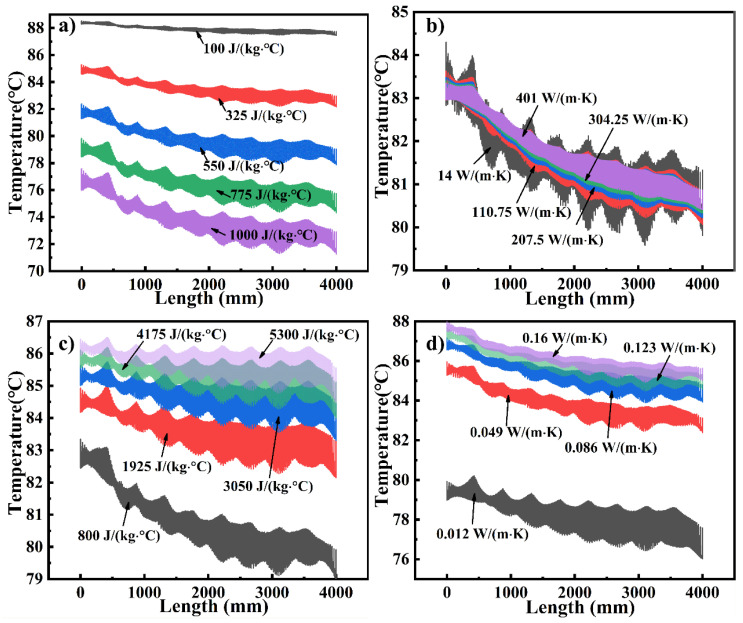
Temperature distribution projection as a function of (**a**) specific heat of the mold, (**b**) thermal conductivity of the mold, (**c**) specific heat of the fluid, and (**d**) thermal conductivity of the fluid.

To describe the temperature distribution on the mold panel, all the temperature data at t = 300 min were projected along the length of the mold (Figure 14). Clearly, the temperature on the windward side of the mold is higher than that on the leeward side. As the specific heat of the mold decreases and the specific heat and thermal conductivity of the fluid increase, the T_avg_ increases, and the temperature uniformity of the mold improves, as observed from the height of the temperature region. The thermal conductivity of the mold has a significant influence on the temperature uniformity but only a minor effect on the T_avg_ and average heating rate. These conclusions corroborate the ones stated above.

**Figure 15:**
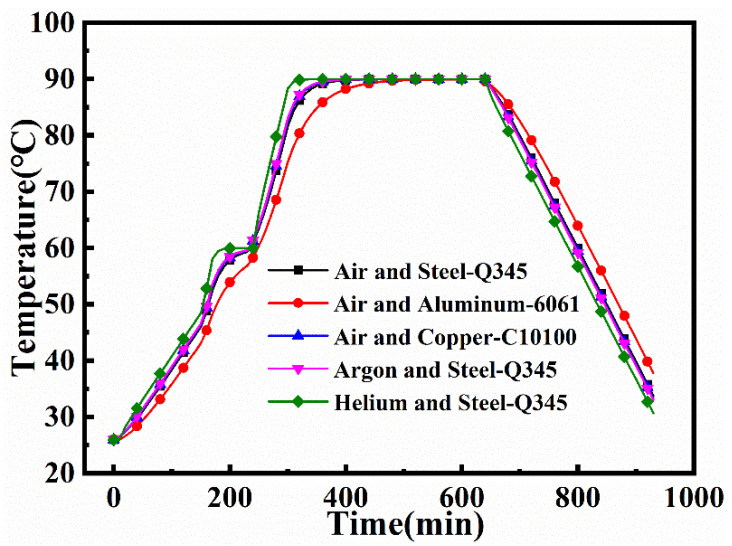
Average temperatures of the mold panel.

Figure 15 shows the T_avg_ of the mold panel for combinations of five different fluid and mold materials. In the heating stage, when air was used as the heat transfer medium, t = 300 min, the T_avg_ of the steel, copper, and aluminum molds are 81.64 °C 82.64 °C, and 75.29 °C, respectively, and their heating rates are 0.348 °C /min, 0.360 °C/min, and 0.285 °C/min, respectively. In the heating stage, when helium is taken as the heat transfer fluid, the T_avg_ and heating rate of the steel mold are higher than those in the air. This is mainly because the thermal conductivity and specific heat capacity of helium are higher than those of air. When argon serves as the heat transfer material, the T_avg_ and heating rate of the steel mold are also higher than those in air. This is because although the thermal conductivity of argon is lower than that of air, its specific heat capacity is higher. At the time t = 300 min, the T_avg_ of the steel mold panel in helium and argon are 88.35 °C and 83.08 °C, respectively, and their heating rates are 0.462 °C/min and 0.365 °C/min, respectively.

**Figure 16:**
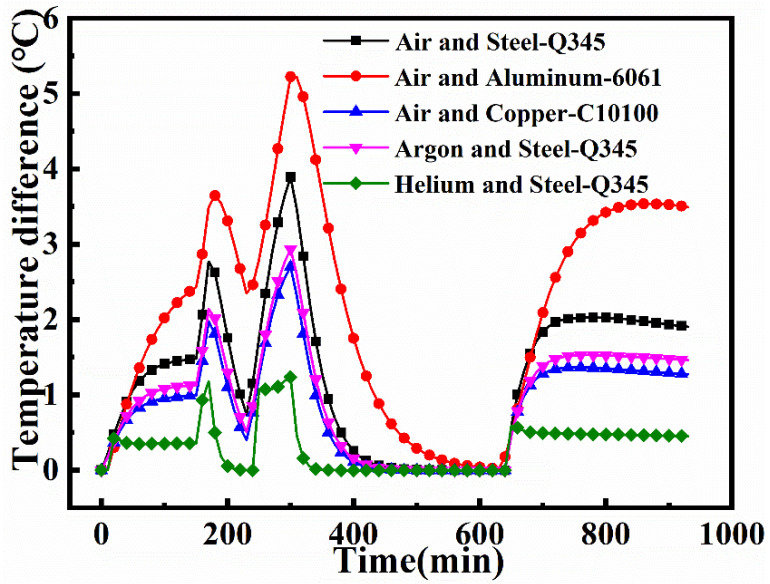
Maximum temperature difference on the mold plate.

Figure 16 shows the TD_max_ for a large framed mold, for combinations of five different fluid and mold materials. When air is taken as the heat transfer fluid in both the heating as well as cooling stages, the TD_max_ for the aluminum mold is larger than that for the steel mold, while the TD_max_ for the copper mold is smaller than that for the steel mold. This is mainly because the thermal conductivity of copper is higher than that of steel, and the specific heat capacity is lower. The TD_max_ for the mold panel is the highest at the ending time of the heating phase (t = 300 min). The maximum TD_max_ that a steel, aluminum, and copper molds reaches is 3.89 °C, 5.22 °C (an increase of 34.16% over that of the steel mold), and 2.70 °C (30.63% lower than that of the steel mold), respectively. When helium is applied as the heat transfer material, the TD_max_ for the mold is relatively low in both the heating as well as cooling stages. This is because the thermal conductivity and specific heat capacity of helium are higher than those of air. When argon serves as the heat transfer material, the TD_max_ for the mold is also lower than that air is taken as the heat transfer fluid. Although the thermal conductivity of argon is lower than that of air, its specific heat capacity is higher. It is clear that the specific heat capacity plays a key role in the temperature distribution. At t = 300 min, the maximum TD_max_ for the steel mold using helium and argon as the medium are 1.24 °C and 2.94 °C, respectively. These values are 68.27% and 24.55% lower, respectively, than those with air as the medium.

**Figure 17:**
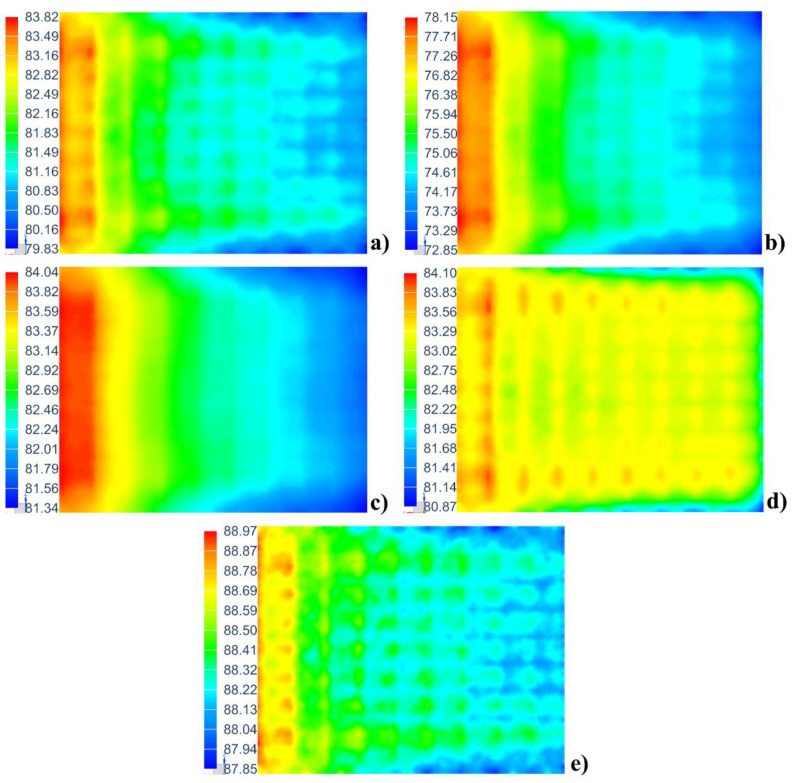
Temperature contour plot of the mold panel (unit: °C): (**a**) steel-Q345 and air, (**b**) aluminum-6061 and air, (**c**) copper-C10100 and air, (**d**) steel-Q345 and argon, and (**e**) steel-Q345 and helium.

The temperature contour plots at t = 300 min for a large framed mold for combinations of five different fluid and mold materials are shown in Figure 17. The temperature distribution on the mold plate is the result of the combined effect of the thermal parameters of the materials and the mold structure. The molds used in the five combinations are provided with the same structure. As shown in Figure 17, the five combinations have a similar temperature distribution trend, in that the temperatures close to the windward side (left) are significantly higher (the red represents high temperature and the blue represents low temperature) than those close to the leeward side (right). The difference between the five combinations is reflected in the temperature range. In Figure 17b, the temperature range of the combination of air and aluminum-6061 is 72.85–78.15 °C, which is larger than that of other combinations, though the temperature is not high. Additionally, it is obvious that the temperature contour plots of the five combinations are different. The above is the influence of thermal parameters of different fluid and mold materials on the temperature field distribution.

**Figure 18:**
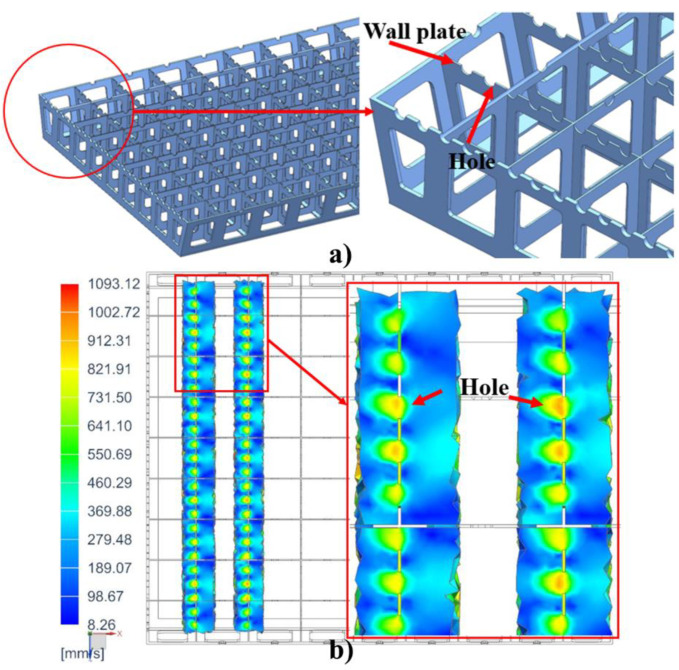
(**a**) Supporting frame structure of mold; (**b**) Fluid velocity distribution at the hole (unit: mm/s).

The mold structure has an effect on the temperature distribution on the mold plate because the supporting frame structure of the mold is composed of many wall plates with holes (Figure 18a). When the fluid flows through a hole, the flow velocity at the hole is relatively high (Figure 18b), so the heat exchange efficiency is higher, and the temperature of the mold plate corresponding to the hole is higher.

**Figure 19:**
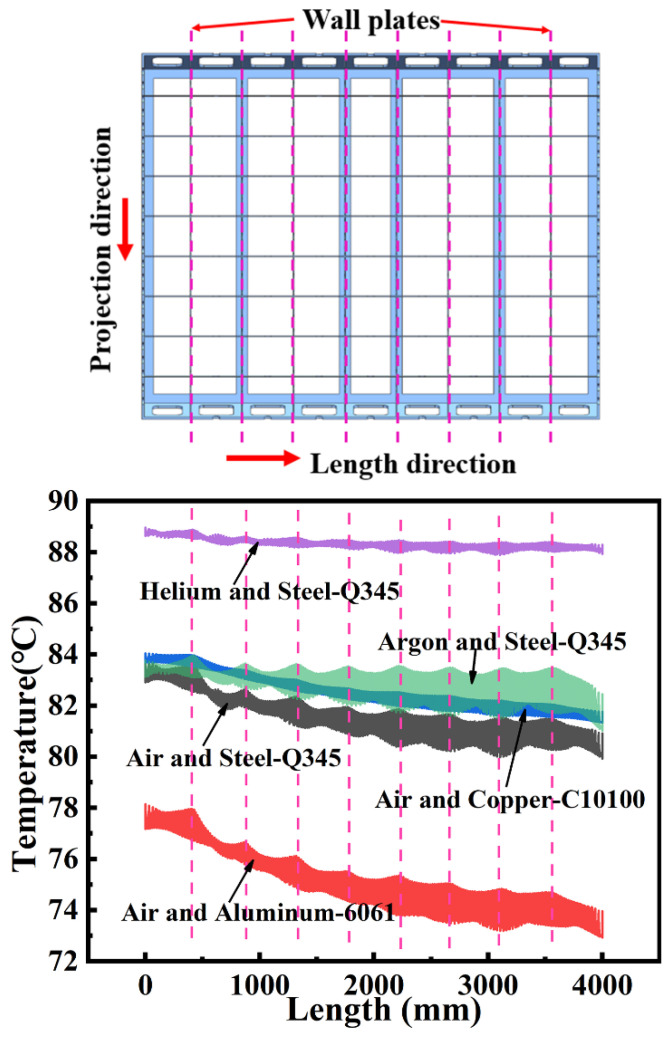
Temperature distribution projection and corresponding wall plates.

Only the temperature distribution trend and temperature range for a large framed mold for combinations of five different fluid and mold materials can be observed in Figure 17. In order to more intuitively compare the temperature performance of the five combinations, all the temperature data on the mold panel (in Figure 17) at t = 300 min are projected to the length direction of the mold, as shown in Figure 19. There are five areas corresponding to the combinations of the five different fluid and mold materials: the smaller the area, the more uniform the temperature field distribution on the mold panel. The area of the combination of helium and steel-Q345 (purple) is the smallest and its temperature is the highest; therefore, the combination has a good temperature performance. Conversely, the area of the combination of air and aluminum-6061 (red) is the largest and its temperature is the lowest; thus, the combination has a poor temperature performance. The edge of the area is not smooth, as shown in Figure 19. The protrusion on the edge of the area corresponds to the position of the wall plates of the mold. The location of the protrusion indicates a high temperature. The temperature here is the temperature of the corresponding mold panel above the hole. The high temperature is mainly caused by the high flow velocity at the hole. Comparing three different metal molds, the copper mold has an advantage on temperature performance, which is slightly better than that of the steel mold, while the aluminum mold shows poor temperature performance. Therefore, copper and steel are suitable materials for large framed molds. As heat transfer fluids, helium and argon can improve the temperature performance of large frame molds. Hence, helium and argon can be mixed with air and used as heat transfer media in autoclaves for improving the temperature performance of industrial large framed molds to ensure the optimum quality of large composite parts.

## 6. Conclusions

In this study, a CFD simulation model was developed for a large framed mold, taking into account the convective heat transfer and turbulent flow inside the autoclave. The model was verified by the data from a curing experiment, in which the temperature data in a large framed mold panel was monitored. The CFD model can accurately predict the temperature field distribution of the large framed mold and the fluid–thermal–solid interaction inside the autoclave.

By combining the experimentally verified CFD model and the single-variable method, numerical simulations were performed for studying the influence of mold and fluid thermal parameters on the heating performance, including the heating rate and temperature uniformity of the large framed mold. The results show that the mold and fluid thermal parameters significantly influenced the heating performance of the mold. Our model predicts that using a mold material with low specific heat capacity or high thermal capacity and/or a fluid with high specific heat capacity and thermal conductivity would improve the heating rate and temperature uniformity of the mold. The results were verified using combinations of five different mold and fluid materials. Hence, to manufacture large framed molds, steel and copper are preferable for enhanced temperature field distribution. Additionally, gases with high specific heat capacities, such as helium and argon, can be mixed with air and used in the autoclave as heat transfer media to improve the heating performance of the large framed mold and ensure optimum quality of large composite parts.

## Figures and Tables

**Table 1:** Thermal properties of metals considered in this study.

Metal	Metal Grade	Density	Thermal Conductivity	Specific Heat Capacity
		g/cm^3^	W/(m∙K)	J/(kg∙°C)
	Q345 (China)	7.829	54.6	434
	AISI 310	7.93	14	500
	AISI 410	7.73	24.2	460
Steel	AISI 304	7.90	16.3	500
	AISI 1005	7.87	56	481
	AISI 1008HR	7.87	65.2	481
	AISI 4340	7.85	44.5	475
	2014	2.79	154.25	880
Aluminum	5086	2.66	117	900
	6061	2.71	154.25	896
	A356	2.67	151	963
Copper	C10100	8.92	387	385

**Table 2:** Thermal properties of gases considered in this study.

Fluid	Density	Thermal Conductivity	Specific Heat Capacity
	kg/m^3^	W/(m∙K)	J/(kg∙°C)
Air	1.2041	0.0263	1007
Argon (Ar)	1.78	0.0154	2951
Carbon dioxide (CO_2_)	1.5362	0.02047	900
Nitrogen (N_2_)	1.142	0.0262	1042
Oxygen (O_2_)	1.3007	0.02676	920.3
Helium (He)	0.1625	0.152	5193

**Table 3:** Collected data of all numerical simulations.

Specific Heat Capacity of the Mold	Thermal Conductivity of the Mold	Specific Heat Capacity of the Fluid	Thermal Conductivity of the Fluid
J/(kg∙°C)	W/(m∙K)	J/(kg∙°C)	W/(m∙K)
100–1000	54.6	1007	0.0263
434	14–401	1007	0.0263
434	54.6	800–5300	0.0263
434	54.6	1007	0.012–0.16

**Table 4:** Combinations of metal and fluid.

Case	Mold	Fluid
1	Steel-Q345	Air
2	Aluminum-6061	Air
3	Copper-C10100	Air
4	Steel-Q345	Argon
5	Steel-Q345	Helium

## Data Availability

No new data were created or analyzed in this study. Data sharing is not applicable to this article.
